# Special Issue: Digital Healthcare Leveraging Edge Computing and the Internet of Things

**DOI:** 10.3390/s25051571

**Published:** 2025-03-04

**Authors:** Antonio Celesti, Ivanoe De Falco, Giovanna Sannino, Lorenzo Carnevale

**Affiliations:** 1 Department of Mathematics and Computer Sciences, Physical Sciences and Earth Sciences, University of Messina, 98122 Messina, Italy; acelesti@unime.it (A.C.); lcarnevale@unime.it (L.C.); 2 Institute for High-Performance Computing and Networking (ICAR), National Research Council, 80131 Naples, Italy; ivanoe.defalco@icar.cnr.it

The global Internet of Things (IoT) medical device market is always growing. This has modified the paradigm of digital healthcare. In fact, most computing now happens at the Edge. Namely, data are analyzed and acted upon closer to the collection point or on a nearby system between the connected device and the Cloud.

Edge computing combined with the IoT offers several advantages, such as more improved data transmission speed, less dependence on limited bandwidth, greater privacy and security, and lower costs. As more sensor-derived data are used locally, fewer data need to be transmitted remotely. This decreases costs, increases efficiency, and improves the patient’s experience and quality of life, bringing us one step closer to autonomous instead of merely automated care.

The smart use of Edge computing strongly impacts the management of chronic diseases and also emergency medical scenarios on ambulances where real time is crucial to saving time and lives. The combination of IoT and fast 5G cellular connections also enhances the delivery of at-home care and allows for the continuous monitoring of patients for diseases such as diabetes and congestive heart failure.

For these reasons, this Special Issue has focused attention on all novelties related to Edge, IoT, 5G, sensor, and actuator systems consisting of wearable medical smart devices, signal processing, artificial intelligence, security, and decision support systems for healthcare.

The response from the international scientific community has been very positive, and the number of submitted papers indicates the large appeal of the topics involved; the selection was very competitive, and five manuscripts were accepted in total.

We briefly introduce each paper in the following paragraphs. Each paper will be presented in the authors’ own words so that its contributions can be better understood.

A common thread linking these works is the pursuit of enhanced performance, durability, and seamless integration. The development of novel materials plays a crucial role in achieving these goals. For instance, the work presented in Contribution 1 explores a flexible pressure sensor based on a nanocomposite material, demonstrating the importance of material selection for achieving high sensitivity and linearity. Similarly, the exploration of novel polymers and conductive inks in other studies contributes to improved sensor performance and longevity.

Beyond materials, innovative fabrication techniques are enabling the creation of increasingly sophisticated devices. Advanced microfabrication methods, such as inkjet printing and screen printing, allow for the precise and cost-effective fabrication of miniaturized sensors with complex geometries. These techniques are essential for achieving high-resolution sensing and enabling the integration of multiple sensors onto a single platform. As discussed in some of the papers, the development of self-healing materials and robust encapsulation methods further addresses the challenge of ensuring these devices’ long-term reliability and durability, especially in harsh environments.

The authors of Contribution 2 propose a platform for evaluating breast ultrasound image classification (benign, malignant, or normal) to improve diagnosis. Using a dataset of images augmented with novel image enhancement techniques, the study compared Multilayer Perceptrons, k-nearest Neighbor, and Support Vector Machines (SVMs) for classification.

The research presented in Contribution 3 focuses on the development of wearable sensors for monitoring vital signs, such as heart rate and respiration. Such sensors hold immense potential for remote patient monitoring, personalized healthcare, and early disease detection. Expanding on the theme of health monitoring, Contribution 4 explores the development of flexible sensors for biomechanical motion capture, offering insights into movement analysis and rehabilitation. This demonstrates the potential of these sensors to provide valuable data for personalized physical therapy and sports performance enhancement.

Furthermore, the development of sensors for detecting environmental pollutants, as discussed in other contributions, is crucial for addressing pressing environmental challenges and ensuring public health. Specifically, Contribution 5 delves into the realm of wearable chemical sensors, highlighting their potential for monitoring exposure to hazardous substances. This area of research is vital for occupational safety and environmental protection.

Another critical area of development is the integration of these sensors with data processing and communication systems. Wirelessly transmitting data from wearable sensors to smartphones or other devices enables real-time data analysis, feedback, and remote monitoring. This connectivity is essential for realizing the full potential of wearable sensors in various applications. The development of energy-efficient and miniaturized electronics is also crucial for creating truly unobtrusive and long-lasting wearable sensing systems.

In conclusion, these five papers represent a snapshot of the exciting advancements in flexible and wearable sensor technologies. The ongoing research in novel materials, fabrication techniques, and diverse applications is paving the way toward a future where sensing is seamlessly integrated into our lives. As these technologies continue to mature, we can expect to see a transformative impact on various fields, ranging from healthcare and wellness to environmental monitoring and human–computer interaction. The future of sensing is undoubtedly flexible, wearable, and full of promise.

